# Outpatient Management of Operative Ankle Fractures and Its Benefits: A Predictive Model for Patient Selection

**DOI:** 10.7759/cureus.114361

**Published:** 2026-08-11

**Authors:** Derek R Bass, Parth A Goenka, William F McCormick, Nicole H Lewis, Robert M Harris

**Affiliations:** 1 Orthopedic Surgery, East Tennessee State University, Johnson City, USA; 2 Quillen College of Medicine, East Tennessee State University, Johnson City, USA; 3 Trauma and Orthopedics, The Hughston Clinic, Columbus, USA

**Keywords:** ankle, foot, fracture, orthopedic, trauma

## Abstract

Background

Outpatient management of operative ankle fractures has consistently demonstrated financial and clinical benefits in the literature. We aim to determine factors associated with preoperative and postoperative complications of ankle fractures and create a predictive model that will assist surgeons in identifying patients optimized for outpatient operative management.

Methods

We performed a retrospective analysis of adult patients presenting to a single Level I trauma center from 2019 to 2024 with ankle fractures requiring open reduction and internal fixation (ORIF). The primary outcome was any complication, and secondary outcomes were preoperative complications and postoperative complications. Complications were then compared to patient characteristics using univariate analyses, followed by multivariate logistic regression to identify independent predictors of complications.

Results

A total of 318 patients were included, with 78 experiencing one or more complications. Time to surgery, kidney disease, diabetes, drug use, chronic obstructive pulmonary disease, cardiac comorbidities other than heart failure, and nicotine use were significantly associated with complications either prior to or following operative fixation. Time to surgery, diabetes, and drug use were associated with preoperative complications. When screening for the likelihood of preoperative complications, model performance demonstrated adequate discrimination and calibration, with an area-under-the-curve of 0.78, balanced accuracy of 0.75, sensitivity of 0.86, specificity of 0.64, positive predictive value of 0.20, negative predictive value of 0.98, and a Hosmer-Lemeshow p-value of 0.63.

Conclusion

Time to surgery, recreational drug use, and diabetes were found to be significantly associated with preoperative complications. The resulting model can reliably identify patients at low risk of preoperative complications with a sensitivity of 0.86and a negative predictive value of 0.98. Treating physicians may utilize this model to select patients optimized for safer outpatient management.

## Introduction

Ankle fractures are among the most common orthopedic injuries, accounting for approximately 10% of musculoskeletal injuries annually [1]. When requiring open reduction and internal fixation (ORIF), ankle fractures have traditionally been managed with inpatient admission. However, the growing body of literature suggests that outpatient management of ankle fractures offers significant economic, clinical, and patient-centered advantages over traditional inpatient care, particularly for patients with isolated, uncomplicated fractures.

Cost and resources

Inpatient care of ankle fractures has been demonstrated to be associated with a significant economic burden estimated at $367 million in excess annual costs when compared to outpatient management [1]. This finding is consistent with those of other studies, which reported a 50% reduction in costs for outpatient surgeries ($ 1,834 vs. $ 4,137 per case) and a nearly $ 9,000 reduction in 90-day costs per case ($12,923 vs. $21,866) [2,3]. The shorter hospital stay, 7.5 hours for outpatients versus 54.3 hours for inpatients, was the primary driver of these savings [3].

Outpatient management has also been shown to conserve healthcare resources. By decreasing patient load on the inpatient service, hospitals are better able to preserve necessary personal protective equipment and hospital beds while also enabling more efficient use of operating rooms and post-operative care resources [3-5]. 

Clinical outcomes

Various studies have demonstrated significantly lower rates of both postoperative and clinical complications with outpatient management of fractures. Complications, including urinary tract infections and venous thromboembolic events, have been shown to occur less frequently with outpatient management [6]. The risk of infection for inpatient foot and ankle procedures was found to be nearly 10 times higher than for corresponding outpatient management (15.9% vs. 1.7%) [7]. Outpatient management is also associated with a significantly decreased time to weight-bearing and fewer opioid prescriptions, enhancing overall patient satisfaction [2,4,5].

A variety of factors, including fracture type and pattern, health status, and social history, have been associated with complications following operative management of ankle fractures [8-10]; however, inpatients are overall 2.14 times more likely to develop major complications, even when controlling for comorbidities [11]. 

In summary, outpatient care leads to reduced healthcare costs, fewer complications, and quicker recovery times, without compromising patient safety. However, further research is needed to identify subgroups of patients who may still benefit from inpatient care, such as those with complex fractures or multiple comorbidities. The purpose of this study was to evaluate the risks of complications for outpatient versus inpatient treatment of operative ankle fractures and to identify factors that may affect pre- and postoperative outcomes.

## Materials and methods

Data collection

The study was conducted at the Johnson City Medical Center in Johnson City, TN, USA. The East Tennessee State University IRB issued approval 1024.6e for a retrospective data analysis of patients with surgically managed ankle fractures. Patients were identified by current procedural terminology (CPT) codes (Table 1), and characteristics (sex, race, age, BMI, substance use, comorbidities), injury data (classification, mechanism, time to surgery), and outcome data (preoperative complications, postoperative complications, and readmissions) were collected. 

**Table 1:** Current procedural terminology (CPT) codes used to identify patients and a description of their corresponding procedures.

CPT Code	Procedure Description
27766	Percutaneous treatment of distal fibular (lateral malleolus) fracture with manipulation
27769	Percutaneous treatment of distal fibular (lateral malleolus) fracture without manipulation
27784	Open treatment of the distal fibular (lateral malleolus) fracture (ORIF)
27792	Open treatment of the distal tibiofibular joint (syndesmosis) disruption
27814	Open treatment of the distal tibial (medial malleolus) fracture
27822	Open treatment of a bimalleolar ankle fracture
27823	Open treatment of trimalleolar ankle fracture
27829	Open treatment of trimalleolar ankle fracture

The primary outcome of this study was the occurrence of any complication, defined as a medical or surgical adverse event, related to the injury or its treatment, occurring before surgery or within 90 days after. Only patients with operatively treated ankle fractures were included. Patients less than 18 years old, with follow-up less than 90 days, or incomplete data were excluded. The Strengthening the Reporting of Observational Studies in Epidemiology (STROBE) guidelines were used to ensure proper development and documentation of this study.

Statistical analysis

Factors associated with pre-surgical, post-surgical, and overall complications following ankle fracture surgery were evaluated. Demographic variables, comorbidities, substance use indicators, and surgical characteristics were examined as potential predictors. For pre-surgical complications, a multivariable logistic regression model was developed using stepwise selection based on Akaike’s Information Criterion to identify the most parsimonious and significant set of predictors. Model performance was assessed through standard diagnostics, including goodness-of-fit testing, discrimination metrics, calibration, and evaluation of clinical utility using a decision curve analysis. Since the outcome was uncommon, the Youden Index was used to determine the best threshold value to optimize sensitivity and specificity.

Post-surgical complications and overall complications were examined using bivariate analyses. Independent two-sample t-tests were used to compare continuous variables, and Fisher’s exact tests were used for categorical variables when assumptions for chi-square testing were not met. Significance was defined at the α = 0.05 level. 

## Results

Patient demographics

A total of 547 patients were identified by CPT code, of which 229 were excluded on the basis of incomplete data or inadequate follow-up. Of the 318 patients included in the study, 222 were female (69.8%), and 96 were male. The average patient age was 50.32 years, with a mean body mass index of 32.12. The mean time from injury to surgery was 11.09 days, and the average operative time was 85.03 minutes. Fracture patterns included 93 trimalleolar fractures, 93 bimalleolar fractures, 83 bimalleolar-equivalent fractures, 37 lateral malleolus fractures, and 12 medial malleolus fractures. In regard to race, 306 patients identified as White (96.2%), eight as Black, and four as Hispanic individuals. Nicotine use was reported by 116 patients; recreational drug use was reported by 40 patients, and some degree of alcohol use was reported by 100 patients.

Overall, 78 patients experienced a total of 87 complications, with nine patients experiencing both preoperative and postoperative complications. In all, 25% of medial malleolar (3/12), 29.7% of lateral malleolar (11/37), 22.9% of bimalleolar equivalent (19/83), 24.7% of bimalleolar (23/93), and 23.7% of trimalleolar fractures (22/93) led to a complication. There were 29 preoperative complications, occurring at a mean of 6.55 days following injury, with an associated mean time to surgery of 17.5 days. Additionally, 58 postoperative complications were observed, with a mean time to surgery of 11.56 days and a mean time to complication of 32.7 days after surgery.

Preoperative complications

The stepwise logistic regression model identified time to surgery, recreational drug use, and diabetes as significant predictors of pre-surgical complications. Longer time between injury and surgery was associated with increased risk (OR = 1.08 per day delay), and both recreational drug use (OR = 2.77) and diabetes (OR = 2.39) were independently associated with pre-operative complications. These three significant variables were used to create a predictive model, which is described below: x = time to surgery in days; y = presence (1) or absence (0) of drug use; z = presence (1) or absence (0) of diabetes. Step 1: Calculate linear predictor (*n) **n = -3.722 + (0.077x) + (1.018y) + (0.870z)*; Step 2: Convert *n *to probability (p) $p=1/(1+e^{-n})$; Step 3: Compare probability (p) to threshold (0.0719) $p\ge0.0719\to No Complication$ *p< 0.0719→ Potential Complication*

This model demonstrated good overall performance, exhibiting good model fit (Hosmer-Lemeshow p = 0.63), no evidence of multicollinearity, and acceptable discrimination (AUC = 0.78). Calibration metrics indicated strong agreement between predicted and observed risks. 

Since pre-surgical complications were uncommon, classification was based on the Youden Index threshold, which yielded a sensitivity of 86% and a specificity of 64%. The model also demonstrated a high negative predictive value (98%), reflecting a strong ability to identify patients at low risk for complications; however, the positive predictive value remained modest due to the rarity of the outcome. Decision curve analysis showed that the model provided a net clinical benefit relative to treat-all and treat-none strategies across threshold probabilities between 5% and 20%, supporting its potential use as a preoperative risk stratification tool.

Postoperative complications

Independent two-sample t-tests were performed for each numerical variable (time to surgery, length of surgery, age at injury, height, weight, and BMI), and found no significant differences in the mean response between the two groups for the presence of postoperative complications.

Fisher’s exact tests were conducted to examine the association between postoperative complications and each categorical variable individually. Significant associations were observed for chronic kidney disease (p = 0.0004), diabetes (p = 0.0316), and nicotine use (p = 0.0495). No other categorical variables demonstrated statistically significant relationships with postoperative complications (Table 2).

**Table 2:** Results of tests comparing patient groups created based on the presence or absence of a postoperative complication. The t-tests for continuous variables and Fisher’s exact test for categorical variables for postoperative complications. Significant p-values are in bold.

Postoperative complications	
t-test	Result
Time to Surgery	p=0.597
Length of Surgery	p=0.169
Age at Injury	p=0.432
Height	p=0.789
Weight	p=0.889
BMI	p=0.985
Fisher’s Exact Test	
Chronic Kidney Disease	p=0.000371
Diabetes	p=0.0316
Nicotine Use	p=0.0495
Chronic Obstructive Pulmonary Disease (COPD)	p=0.105
Pulmonary Comorbidities (other than COPD)	p=0.105
Recreational Drug Use	p=0.124
Movement Disorder	p=0.217
Heart Failure	p=0.242
Cardiac Comorbidities (other than heart failure)	p=0.282
Alcohol Use	p=0.327
Gastrointestinal Comorbidities	p=0.351
Ethnicity	p=0.570
Malignancy	p=0.798
Fracture Classification	p=0.800
Sex	p=0.875
Endocrinopathies	p=1.0

Overall complications

Independent two-sample t-tests were performed for each numerical variable (time to surgery, length of surgery, age at injury, height, weight, and BMI), and found a significant difference in mean time to surgery, which was significantly less (10.475 days) for those without a complication compared to those with any complication (13 days; p=0.009351). There were no other statistically significant differences between the two groups for the five remaining variables. 

Results of Fisher’s exact tests showed significant associations between any complication and multiple comorbid conditions, including kidney disease (p = 0.0003), diabetes (p = 0.0056), chronic obstructive pulmonary disease (p = 0.0117), and other cardiac comorbidities (p = 0.0183), as well as nicotine use (p = 0.0435) and recreational drug use (p = 0.0094). Results for all variables are shown in Table 3. 

**Table 3:** Results of tests comparing patient groups created based on the presence or absence of any complication. The t-tests were used for numerical values and Fisher’s exact test for categorical values for all complications. Significant p-values are in bold.

Overall complications	
t-test	Result
Time to Surgery	p=0.00935
Length of Surgery	p=0.954
Age at Injury	p=0.999
Height	p=0.718
Weight	p=0.931
BMI	p=0.901
Fisher’s Exact Test	
Chronic Kidney Disease	p=0.000284
Diabetes	p=0.00560
Recreational Drug Use	p=0.00943
Chronic Obstructive Pulmonary Disease (COPD)	p=0.0117
Cardiac Comorbidities (other than heart failure)	p=0.0183
Nicotine Use	p=0.0435
Heart Failure	p=0.176
Ethnicity	p=0.201
Alcohol Use	p=0.201
Pulmonary Comorbidities (other than COPD)	p=0.210
Movement Disorder	p=0.266
Gastrointestinal Comorbidities	p=0.406
Malignancy	p=0.646
Sex	p=0.673
Fracture Classification	p=0.941
Endocrinopathies	p=1.0

## Discussion

Our results present similar findings to the established literature regarding inpatient versus outpatient management for ankle fractures. Numerous studies have demonstrated that outpatient management is associated with lower complication rates and improved efficiency of care compared with inpatient admission, even when controlling for age and comorbidity burden [7,11]. These same studies found that inpatient management presents up to twice the risk of major complications, including infection and thromboembolic events, compared to outpatients, with diabetes and age-related comorbidities frequently implicated as key drivers of risk [7,11].

Our analysis found diabetes to be significantly associated with both preoperative and postoperative complications, as well as overall complication risk. This parallels findings from prior studies demonstrating markedly increased infection-related complications in diabetic ankle fracture patients [9,12-14], emphasizing the importance of tailored perioperative management in this population. Additionally, kidney disease was independently associated with postoperative complications in our cohort, further emphasizing the importance of systemic disease as a factor.

Logistic regression identified increasing time to surgery as being significantly associated with preoperative complications, and overall complications were significantly more common when time to surgery exceeded approximately 10 days. These findings strongly align with published literature showing that delays in surgical fixation increase total episode-of-care costs and complication rates [6,15]. Accordingly, our results support the growing argument for rapid outpatient scheduling pathways that decrease the preoperative interval and expedite definitive fixation.

Importantly, while many national datasets emphasize comorbidity-driven risk, our findings highlight patient behaviors as well. Nicotine use was associated with postoperative complications, while recreational drug use was associated with both preoperative and overall complication risk. These findings emphasize the importance of incorporating psychosocial and behavioral factors into risk-stratification algorithms in order to improve accuracy and optimize operative outcomes.

A key contribution of our study is the strong discriminative power of our preoperative complication prediction model, which achieved a negative predictive value of 98% and sensitivity of 86%, indicating good predictive capability when identifying low-risk patients who can be safely discharged for outpatient management. While prior literature suggests that healthier and younger patients demonstrate superior outcomes [8-10], the existing literature has lacked a validated preoperative screening tool allowing clinicians to objectively identify which patients can safely undergo same-day discharge and perioperative management. Our model provides a practical solution that aligns with the outpatient care advantages described in the literature, including shorter anesthesia exposure, lower infection rates, lower cost, and reduced systemic complication risk.

Additionally, by rearranging the predictive model to assume outpatient management, we are able to define the maximum time to surgery before a patient is recommended for inpatient management. The recommended times for surgery are then based on the two remaining inputs, diabetes and recreational drug use (Table 4). Patients with both diabetes and drug use are recommended for inpatient management regardless of the time to surgery. This itself can be utilized for medical decision-making in areas less able to accommodate a flexible operating schedule.

**Table 4:** The maximum recommended time for outpatient surgery. Patients with both diabetes and recreational drug use are recommended for inpatient management, even with a “time to surgery” of zero days, and so have no recommendation for time to outpatient surgery.

		Diabetes	
		Yes	No
Drug Use	Yes	n/a	<2 days
	No	<4 days	<16 days

Interestingly, our postoperative complication analysis did not find significant associations with BMI, age, or surgical duration. This diverges from some published work, where operative duration and age are emphasized as predictors of postoperative complication risk, particularly in geriatric cohorts [10]. One explanation may be that our institutional surgical times were relatively uniform and tightly protocolized, or that operative technique and standardized perioperative protocols mitigated some of the age-related vulnerability seen in larger nationwide datasets.

Although the results presented here align with the overwhelming consensus in the literature, our study is not without its limitations. Many of these studies, including ours, rely on retrospective data from large databases, which are prone to selection bias [5,11]. For instance, healthier patients with less complex fractures are more likely to undergo outpatient surgery, while patients with higher comorbidities or more severe fractures may be funneled into inpatient care, skewing the comparison. We have attempted to control for this in our analysis by adjusting for available demographic and clinical covariates; however, residual confounding from unmeasured variables remains possible.

The advantages of outpatient management have been repeatedly demonstrated in the literature. The need for more granular, prospective data, especially for high-risk populations, has been reinforced by many studies [9,10], including this one, and warrants further investigation through controlled studies. Future studies should aim to identify specific patient subgroups who could benefit most from inpatient versus outpatient management, especially among elderly and high-comorbidity patients, where complication rates are notably higher.

## Conclusions

Taken together, these findings reinforce the fundamental conclusion of the broader literature: that outpatient management is safe and effective for a substantial proportion of ankle fracture patients, but proper patient selection remains essential. Our data demonstrate that a model incorporating diabetes, recreational drug use, and time to surgery can reliably identify candidates suitable for outpatient treatment, while additional analysis highlights high-risk comorbidities such as kidney disease and obstructive pulmonary disease that may warrant closer inpatient observation. By integrating these local predictive insights with published national datasets, our work provides a pragmatic framework for determining discharge and surgical setting suitability in ankle fracture management.
